# Curcumin and Type 2 Diabetes Mellitus: Prevention and Treatment

**DOI:** 10.3390/nu11081837

**Published:** 2019-08-08

**Authors:** Francesca Pivari, Alessandra Mingione, Caterina Brasacchio, Laura Soldati

**Affiliations:** Department of Health Sciences, University of Milan, Via A. Di Rudinì, 8, 20142 Milan, Italy

**Keywords:** curcumin, type 2 diabetes mellitus, inflammation, antioxidant activity, clinical trials

## Abstract

Type 2 diabetes mellitus (T2DM) is an ensemble of metabolic diseases that has reached pandemic dimensions all over the world. The multifactorial nature of the pathology makes patient management, which includes lifelong drug therapy and lifestyle modification, extremely challenging. It is well known that T2DM is a preventable disease, therefore lowering the incidence of new T2DM cases could be a key strategy to reduce the global impact of diabetes. Currently, there is growing evidence on the efficacy of the use of medicinal plants supplements for T2DM prevention and management. Among these medicinal plants, curcumin is gaining a growing interest in the scientific community. Curcumin is a bioactive molecule present in the rhizome of the *Curcuma longa* plant, also known as turmeric. Curcumin has different pharmacological and biological effects that have been described by both in vitro and in vivo studies, and include antioxidant, cardio-protective, anti-inflammatory, anti-microbial, nephro-protective, anti-neoplastic, hepato-protective, immunomodulatory, hypoglycaemic and anti-rheumatic effects. In animal models, curcumin extract delays diabetes development, improves β-cell functions, prevents β-cell death, and decreases insulin resistance. The present review focuses on pre-clinical and clinical trials on curcumin supplementation in T2DM and discusses the peculiar mechanisms by which curcumin might ameliorate diabetes management.

## 1. Introduction

Diabetes has reached pandemic dimensions, affecting over 400 million people worldwide [1], and it is also becoming relevant in developing countries. Among the huge and heterogeneous number of patients with diabetes, type 2 diabetes mellitus (T2DM) represents the most prevalent form [2]. From the Global Burden of Diseases study (2016), it emerged that T2DM and its complications were responsible for the 22% increase in disability in the last ten years, impacting significantly on public health [3]. In recent years, enormous advances have been made in the field of diabetes prevention and treatment and in glycaemic management, but the prevalence of cardiovascular complications remains an important issue in T2DM patients [4]. Currently, the preferred therapies for T2DM treatment include the use of anti-diabetic drugs, lifestyle intervention (healthy nutrition and daily physical activity) and monitoring of arterial pressure and lipid profile. Effective prevention and treatment are needed to optimally manage diabetes patients. It is well known that T2DM is a preventable disease [5]. A rigorous prevention should be addressed to people with pre-diabetes or impaired glucose tolerance, who are at high risk of T2DM development. Such prevention encompasses lifestyle modifications, such as starting from a healthy diet and daily physical activity.

Currently, there is growing evidence on the efficacy of the use of medical plant supplements for T2DM prevention and management [6,7,8]. Among these medical plants, curcumin is gaining a growing interest in the scientific community. Curcumin is an active molecule present in the rhizome of *Curcuma longa* plant, also known as turmeric. 

The biological and pharmacological effects of curcumin have been described by several in vitro and in vivo studies, and include antioxidant, anti-inflammatory, anti-microbial, cardio-protective, nephro-protective, anti-neoplastic, hepato-protective, immunomodulatory, hypoglycaemic and anti-rheumatic effects [9,10,11,12].

Regarding diabetes, an animal study highlighted that curcumin and its analogues have a mechanism of action similar to that of thiazolidinedione, an anti-diabetic drug, through peroxisome proliferator-activated receptor-γ (PPAR-γ) activation [13]. Thus, curcumin may be effective in the regulation of glycaemia and lipidaemia [14]—factors that play a key role in T2DM treatment. In particular, several randomized controlled trials (RCTs) have been performed to study effects of curcumin on glycaemic and lipid profiles in subjects with pre-diabetes [15,16] or T2DM [17,18,19].

The present review analyses the role of curcumin in T2DM prevention and treatment, focusing on the preclinical and clinical trials.

### 1.1. Diabetes Mellitus and Its Complications

T2DM is one of the most terrible chronic diseases that affect human health. In recent years, in developing countries, there was a 69% increase in the prevalence of diabetes, whereas in developed countries, a 20% increase is expected between 2010 and 2030 [20]. In particular, T2DM is a chronic and metabolic disease characterised by defects in pancreatic insulin secretion and (or) insulin effect on target tissues, generating a persistent state of hyperglycaemia, inducing metabolic alteration, cell-death and inflammation. The increased morbidity and mortality rates related to T2DM are often associated with vascular complications, such as cardiovascular diseases [21], nephropathy [22], retinopathy [23], endothelial dysfunction, dyslipidaemia and an increase of oxidative stress which is also due to inflammation [24]. In fact, diabetes is characterised by a chronic inflammation and insulin resistance due to the enhancement of pro-inflammatory cytokines production by monocytes infiltrate in adipose tissue. This condition is worsened by angiopoietin-like protein 2 (ANGPTL2), highly expressed in macrophages and adipose tissue, because of its role in T-lymphocytes/macrophages activation and accumulation, promoting impaired insulin response. Moreover, high serum ANGPTL2 levels are involved in the development of renal diabetic disease, because of its positive correlation with albumin-to-creatinine ratio and negative correlation with glomerular filtration rate (GFR) [25]. Among microvascular complications approximately 50% of T2DM patients developed a diabetic peripheral neuropathy, a chronic and progressive disorder affecting the peripheral nervous system and characterised by allodynia, pain and paraesthesia symptoms [26], caused by axonal degeneration and segmental demyelination [27]. Moreover, patients with T2DM are at high risk of infections, indirectly caused by diabetic neuropathy, impaired glycaemic control, and immune system dysregulation [28,29]. Diabetic patients are more prone to develop common infections, like cystitis, enteric infections, external otitis, pneumonia, appendicitis and peritonitis as well as rare and severe infections, such as emphysematous pyelonephritis [30,31]. Furthermore, T2DM patients have higher mortality rates for sepsis compared to other individuals [32].

### 1.2. Curcumin: Structure, Biological Activity and Bioavailability

Turmeric plant, commonly utilised in food preparation as a spice (present also in curry powder) and in the scientific community, is recognised as *Curcuma longa*. This type of plant is characterised by orange tuberous rhizomes and is widely cultivated in South East Asia, where it is used as a natural therapeutic remedy for various pathological conditions since ancient times. The peculiar characteristics that have focused scientists’ attention on curcumin as a nutraceutical are the following: the antioxidant and anti-inflammatory activities and the safety of its pharmacological profile [33]. In addition, curcumin has a potential role both in the prevention and treatment of several diseases because of its variety of actions: anti-bacterial, anti-diabetic, anti-viral and anti-cancer activities [34,35]. This mechanism of action on several molecular pathways is due to the peculiar chemical structure of curcumin, able to have huge numbers of molecular targets. Among the biological effects, the inhibition of reactive oxygen species (ROS) production plays a key role, in particular for inflammatory diseases. In turmeric, several compounds coexist, structurally related to curcuminoids, including curcumin as the main active and most important constituent. The main curcuminoids in commercial turmeric extracts are curcumin (≈75%), demethoxycurcumin (≈20%), and bisdemethoxycurcumin (≈5%) [36]. 

Bioavailability represents the major issue in the use of curcumin as a nutritional supplement. In fact, different studies on curcumin bioavailability and pharmacokinetics recognized its low absorption rate in the gastro-intestinal tract and its rapid body elimination [37]. Several animal studies, especially in rodents, have described the rapid metabolism, low absorption, bio-distribution and rapid excretion of curcumin (oral administration) [38,39,40]. For these reasons, recent studies focused on developing new techniques to solve these issues and improve curcumin bioavailability, intestinal absorption and body distribution. These approaches include, for example, micellas, nanoparticles, liposomes and phospholipid complexes [41]. Among these approaches, one of the most promising is phytosome technology, which proved effective in the improvement of curcumin oral bioavailability. Moreover, its particular structure enhanced pharmacokinetic parameters after the oral administration of curcuminoids. Phytosomal curcumin efficacy and tolerability have been proven in the improvement of inflammatory diseases, such as cancer, cardiovascular disease and osteoarthritis. Importantly, there is a body of evidence for the clinical use of phytosomal curcumin in patients with solid tumours, uveitis, benign prostate hyperplasia, diabetic micro-angiopathy and retinopathy [9,42]. Other technologies that showed advantages in increasing polyphenols solubility and bioavailability include food lipid nanoparticles, in particular solid lipid nanoparticles. For example, Sun and colleagues discovered that curcumin lipid nanoparticles could lengthen in vitro cellular uptake and anti-tumour activity and increase the in vivo bioavailability of the loaded curcumin [43]. Moreover, curcumin encapsulation in camel β-casein micelle increased the solubility of curcumin by over 2000-fold [44]. This specific encapsulation influences curcumin antioxidant activity, cytotoxicity in an in vitro model of human leukemia and anti-proliferation activity against in vitro models of human colorectal and pancreatic cancer [45,46].

Various studies also demonstrated an increased oral bioavailability after curcumin nanoemulsion incorporation, facilitating its application as phytochemical in humans [47].

## 2. Curcumin and Type 2 Diabetes Mellitus

### 2.1. Molecular Mechanisms of Curcumin in T2DM

Several studies have shown that curcumin improves the pathologic events in T2DM through different mechanisms and multiple molecular targets (see Table 1). In particular, curcumin is involved in the regulation of lipid metabolism. Curcumin reduces the gene expression of transcription factors involved in hepatic lipogenesis, such as the sterol regulatory element-binding protein 1c (SREBP1c), which promotes cholesterol synthesis and the carbohydrate response element-binding protein (ChREBP) [48]. Moreover, curcumin increases the activity of lipid mobilization enzymes, including carnitine palmitoyltransferase 1 (CPT1) and acyl-CoA cholesterol acyltransferase (ACAT).

In addition, an animal study found that curcumin is involved in the pathological fat accumulation in the liver through the up-regulation of PPAR-γ via AMPK activation [49]. Moreover, the anti-hyperglycaemic and anti-hyperlipidaemic effect of curcumin has been demonstrated in streptozotocin (STZ)-induced diabetic rats after curcumin supplementation (30–90 mg/kg of body weight) in a formulation of yogurt for a period of 31 days [50]. Recently, the same research group described that isolated treatments using curcumin (90 mg/kg of body weight) plus insulin (1 U/day vs. 4 U/day) decreased blood glucose, biomarkers of liver and kidney damage, improved lipid profile and hepatic antioxidants levels [51]. Curcumin supplementation (50 or 100 mg/kg of body weight) in STZ-induced diabetic rats decreased hyperglycaemia and vascular inflammation through the inhibition of MCP-1, IL-6, HbA1c, TNF-α and lipid peroxidation [52]. Moreover, a lot of data have shown that curcumin can improve insulin sensitivity through decreasing glycaemia and dyslipidaemia in high fat-fed rats [53,54,55,56,57,58,59].

Also, oxidative stress has been related to the pathogenesis of T2DM. The protective effect of curcumin against oxidative damage has been proven. Curcumin reduces lipid peroxidation through the normalization of antioxidant enzyme levels, such as superoxide dismutase, catalase and glutathione peroxidase [49]. The administration of tetrahydrocurcumin (80 mg/kg of body weight), a curcumin derivative, for a period of 45 days decreased fasting blood glucose (−55%) and STZ-induced diabetic rats increased their antioxidant defences [60].

Beyond oxidative stress, inflammation also plays a key role in the development of T2DM and its complications. Several studies on animal models of diabetes showed that Curcumin administration decreased the number of inflammatory factors in serum, such as IL-6, TNF-α, MCP-1, and it suppressed the NF-κB signalling pathway, defending against inflammation [21,52]. Also, in vivo studies demonstrated the beneficial effect of curcumin on adipose tissue through the inhibition of several pro-inflammatory mediators, such as MCP-1, IL-1β, TNFα, IL-6 and COX2 [61]. Finally, many authors have confirmed the anti-apoptotic effect of curcumin in diabetic rats with cardiomyopathy. In particular, apoptosis is stimulated in the hearts of rat diabetic cardiomyopathy and curcumin treatment significantly decreased myocardial cell apoptosis [62]. Moreover, curcumin prevents apoptotic and inflammatory process in cardiomyocytes through the inhibition of JNK phosphorylation [63,64]. 

### 2.2. What We Know from Clinical Trials?

Currently, several animal models [65] and human subjects [66,67,68] have shown curcumin tolerability and safety, also at high doses (oral administration of 12 g/day). However, to date it is not recommended to prescribe curcumin supplementation in subjects under particular conditions, such as pregnant or lactating women, children or in patients with anaemia or liver disease due to few safe studies on humans. In animal models, on the other hand, there is a rich scientific literature on oral curcumin safety and tolerability also in delicate conditions [69]. Several in vivo studies have shown oral curcumin safety during pregnancy, also reporting non-mutagenic and non-genotoxic effects [70,71]. Lu and colleagues reported that high curcumin supplementation (100 mg/kg) could also improve glucose and insulin intolerance through activating the AMPK pathway in gestational diabetes mice [72]. Curcumin can be considered as an alternative treatment for gestational diabetes, but further studies are needed. Another open question refers to curcumin interaction with conventional drugs. In fact, some studies identified the effect of curcumin as cytochrome P450 inhibitor [73], conferring potential interaction with anticoagulants, cardiovascular drugs, antibiotics, anti-depressant and anti-cancer drugs [74,75].

Regarding curcumin use in T2DM prevention and treatment, the first data from clinical trials are reported in Table 2. Mohammed et al. recently reviewed published studies focused on the hypoglycaemic action of the spice-derived bioactive ingredients. Despite many of the analysed ingredients showing weak hypoglycaemic effects, curcumin has demonstrated promising hypoglycaemic potential even if further scientific studies are needed to outline safe therapeutic protocols as hypoglycaemic adjuvant [76]. 

A randomized, double-blinded, placebo-controlled trial by Chuengsamarn and colleagues [77] included 240 prediabetic subjects. All subjects were randomly assigned to receive either curcumin (250 mg curcuminoids/day) or placebo capsules for 9 months. After this period, 16.4% of subjects in the placebo group were diagnosed with T2DM, whereas in the group treated with curcumin, none were diagnosed with diabetes. Moreover, in the study group, an improvement in the overall function of β-cells, with lower C-peptide and higher HOMA-β (homeostasis model assessment), emerged. In addition, subjects treated with curcumin, compared to the placebo group, showed higher adiponectin levels and lower insulin resistance. 

Previously, Usharani and colleagues reported an improvement in the antioxidant status, comparable to that of atorvastatin, in T2DM patients supplemented with curcumin capsules (300 mg) for 8 weeks [78].

More recently, Neerati et al. reported that curcumin supplementation (475 mg) for a period of 10 days attenuated hyperglycaemia and hyperlipidaemia in T2DM subjects treated with glyburide. In particular, the low-density lipoprotein, very-low-density lipoprotein and triglycerides were significantly decreased, and the high-density lipoprotein content increased [41]. 

Panahi et al. performed an RCT to assess the effect of nutritional education in combination with curcumin (curcumin 500 mg/day in association with piperine 5 mg/day) or placebo in T2DM patients. A total of 100 T2DM patients aged between 18–65 years were enrolled, and glycaemic, hepatic and inflammatory markers were recorded at baseline and after 3 months. The main results reported a significant reduction in serum levels of C-peptide, HbA1c and glucose in the curcumin-treated group versus the placebo group. Despite expectations, there was no significant difference in the levels of C-reactive protein, an inflammation marker, between the two groups [79]. A previous RCT by the same research group focused on curcuminoid effects on ghrelin, adiponectin, leptin, and TNFα in a cohort of 118 T2DM patients. In particular, curcumin supplementation (curcumin 1000 mg with 10 mg piperine daily) for 12 weeks was effective on adiponectin increase, whereas the leptin/adiponectin ratio (an index of atherosclerosis degree) and leptin levels decreased independently by the weight loss, reflecting a reduction in the inflammatory TNFα marker [80]. The results of these two trials highlighted a beneficial and synergistic effect of curcumin and piperine on glycaemic, inflammatory and hepatic markers in T2DM patients. In particular, piperine seems to significantly improve curcumin intestinal absorption and bioavailability [81].

Na and colleagues performed an RCT to study the effect of curcumin on human blood glucose improvement that they previously found on diabetic rats [57]. One-hundred overweight/obese T2DM patients were randomly divided to the curcumin supplementation (300 mg/day) group or the placebo group for a period of three months. In this trial, the curcumin supplementation group obtained a significant reduction of fasting glycaemia, insulin resistance and HbA1c, together with a decrease in serum triglycerides and total free fatty acids (FFAs), and an improve in lipoprotein lipase activity. In particular, the glucose-lowering effect of curcumin is partially due to a decrease in serum FFA, which may result from promoting fatty acid oxidation and utilisation [18].

## 3. Conclusions

Numerous in vitro and in vivo studies have provided strong evidence for investigating curcumin efficacy against type 2 diabetes mellitus. Data reported in this review show that curcumin has therapeutic potential to counteract diabetes and its complications. All the described studies have shown that doses of up to 12 g per day of curcumin are safe, tolerable and non-toxic. The functional mechanism by which curcumin exerts its effect appears to be the modulation of many signalling molecules. However, this mechanism is not completely clear, due to the complexity of the disease. Based on clinical trials, the clinical efficacy of curcumin seems promising. However, this nutraceutical has poor bioavailability and limited adverse effects reported, representing a major limitation to the therapeutic utility.

Studies on the effect of curcumin on the control of glycaemia and lipid accumulation are lacking, especially in pre-diabetes patients. Moreover, the majority of studies performed tested different preparations of curcumin, especially in combination with other molecules (e.g., piperine), but studies testing pure curcumin are limited. Recently, the results of a meta-analysis have shown the beneficial effects of curcumin and its preparation in Asian pre-diabetes and T2DM patients [7], because most clinical trials were performed on this population. In particular, curcumin appears to be really effective on glycaemic parameters, especially on HbA1c levels. Evaluating all the data in the scientific literature, further clinical trials will be needed to evaluate the effects of curcumin and its specific dosage on glycaemic outcomes, particularly in pre-diabetes and T2DM patients.

## Figures and Tables

**Table 1:** Curcumin and T2DM: molecular and cellular mechanisms discovered in in vivo and in vitro models.

Dosage	Cellular and Molecular Effects	T2DM Prevention and Treatment Potential	In vitro/in vivo Study	Country/Reference
Standard diet with 0.2 g/kg of curcumin for 6 weeks	↓ SREBP1c, ChREBP; ↑ CPT1, ACAT	Regulation of lipid metabolism	diabetic db/db mice	Republic of Korea/[48]
Standard diet with 0.75% of curcumin for 8 weeks	↑ PPAR-γ via AMPK activation; ↓ lipid peroxidation	Anti-oxidant activity	db/db mice	Mexico/[49]
30–90 mg/kg for 31 days	- anti-hyperglycaemic and anti-hyperlipidaemic effect - ↓blood glucose and lipid levels and - ↑the levels of hepatic antioxidants	anti-hyperglycaemic and anti-hyperlipidaemic effect	Streptozotocin-induced diabetic rats	Brasil/[50,51]
100 mg/kg of body weight for 7 weeks; 0.01–1 μM for 24 h	↓ MCP-1, IL-6, HbA1c, TNF-α and lipid peroxidation	Hypoglycaemic, anti-inflammatory and lipid lowering effects	- Streptozotocin-induced diabetic rats - U937 monocytes	USA/[52]
- Curcumin 0.5% w/w; - Curcumin 0.2% plus diet; - 0.05 g/100 g diet; - 80 mg/kg for 60-75 days; - 50, 150, or 250 mg/kg for 7 weeks; - 100 mg/kg for 28 days; - 20 mg/kg for 45 days	↓ glycaemia and dyslipidaemia in high fat-fed rats	Hypoglycaemic effect	Streptozotocin-induced rats fed with high-cholesterol diet (HCD)	Egypt/[53]] India/[54] South Korea/[55] Egypt/[56] China/[57] India/[58] Egypt/[59]
80 mg/kg of body weight for 45 days	↓ blood glucose ↑ antioxidant defences	Hypoglycaemic and anti-oxidant effects	STZ-induced diabetic rats	India/[60]
Curcumin 20 μM	↓ MCP-1, IL-1β, TNFα, IL-6 and COX2	Anti-inflammatory effect	Adipocytes	USA/[61]
200 mg/kg of body weight for 16 weeks	↑ Bcl-2; ↓ Bax, caspase-3	Anti-apoptotic effect	Streptozotocin-induced diabetic rats	China/[62]
- 2.5, 5, or 10 μM - 5 mg/kg once every 2 days for 12 weeks.	↓ JNK phosphorylation	Anti-apoptotic and Anti-inflammatory effect	- Primary cultures of neonatal rat cardiomyocytes; - Streptozotocin-induced diabetic rats	USA/[63] China/[64]

↓ = decrease, ↑ = increase.

**Table 2:** Curcuminoids supplementation in pre-diabetes and T2DM patients clinical trials.

Dosage/Treatment Period	Clinical Trial Type	Study Groups Characteristics	Supplementation Beneficial Effects	Supplementation Adverse Effects	Country/Reference
Curcuminoids: 250 mg/day for 9 months	Randomized, double-blinded, placebo-controlled trial	240 prediabetic subjects: - *n* = 120 placebo group; - *n* = 120 curcuminoids group	- T2DM prevention: 0% T2DM incidence in the treated group vs 16.4% incidence in the placebo group; - β-cells function improvement - ↓C-peptide level; - ↑ HOMA-β level; - ↑ adiponectin level; - ↓ HOMA-IR (insulin resistance).	Major symptoms: none. Minor symptoms: itching (one subject), constipation (two subjects), and vertigo (one subjects)	Thailand/[77]
NCB-02 (curcuminoids): 300mg for 8 weeks	Randomized, parallel-group, placebo-controlled trial	67 T2DM patients: - *n* = 21 placebo group - *n* = 22 atorvastatin group - *n* = 23 NCB-02 group	- endothelial function improvement - ↓ malondialdehyde; - ↓ endothelin-1; - ↓ IL-6; - ↓ TNF-α.	Major symptoms: none. Minor symptoms: mild diarrhoea (two subjects)	India/[78]
Curcuminoids: 475 mg for 10 days	Comparison between glyburide treatment and glyburide plus curcuminoids treatment	8 T2DM patients treated with glyburide (5mg)	↓LDL, VLDL, triglycerides; ↑HDL; glycaemic control imprevement (lower blood glucose levels after breakfast, lunch and dinner)	Major symptoms: none. Minor symptoms: none.	India/[41]
Curcuminoids: 500 mg/day plus piperine 5 mg/day for 3 months	Randomized, double-blinded, placebo-controlled trial	100 T2DM patients: - *n* = 50 placebo group; - *n* = 50 curcuminoids group	- ↓ blood glucose level; - ↓ C-peptide level; - ↓ HbA1c; - ↓ alanine aminotransferase and aspartate aminotransferase.	Major symptoms: none. Minor symptoms: none.	Iran/[79]
Curcuminoids: 1000 mg /day plus 10 mg of piperine/day for 12 weeks	Randomized, double-blinded, placebo-controlled trial	100 T2DM patients: - *n* = 50 placebo group; - *n* = 50 curcuminoids group	- ↓ leptin; - ↓ TNF-α; - ↓ leptin:adiponectin ratio; - ↑ adiponectin.	Major symptoms: none. Minor symptoms: none.	Iran/[80]
Curcuminoids: 300 mg/day for 3 months	Randomized, double-blinded, placebo-controlled trial	100 overweight/obese T2DM patients: - *n* = 50 placebo group; - *n* = 50 curcuminoids group	- ↓ fasting glycaemia; - ↓ HOMA-IR (insulin resistance); - ↓ HbA1c; - ↑ lipoprotein lipase activity; - ↓ FFA and triglycerides.	Major symptoms: none. Minor symptoms: none.	China/[18]

↓ = decrease, ↑ = increase.
